# Double purse-string suture technique for circular-stapled anastomosis during robotic Ivor Lewis esophagectomy

**DOI:** 10.3389/fsurg.2022.957093

**Published:** 2022-07-27

**Authors:** Hanlu Zhang, Zeping Zuo, Xiuji Yan, Fuqiang Wang, Lin Yang, Guanghao Qiu, Long-Qi Chen, Yun Wang

**Affiliations:** ^1^ Department of Thoracic Surgery, West China Hospital of Sichuan University, Chengdu, China; ^2^ Department of Anesthesiology, Laboratory of Anesthesia and Critical Care Medicine, National-Local Joint Engineering Research Center of Translational Medicine of Anesthesiology, West China Hospital of Sichuan University, Chengdu, China; ^3^ Department of Plastic and Burns Surgery, West China Hospital of Sichuan University, Chengdu, China

**Keywords:** esophagectomy, intrathoracic anastomosis, robot-assisted surgery, esophageal cancer, circular-stapled anastomosis

## Abstract

**Background:**

With the advantage of the robotic suturing capacity, the purse-string suture is technically simple and convenient. This study aimed to present our technical aspects and initial results of robotic Ivor Lewis esophagectomy using two purse-string sutures for circular-stapled anastomosis.

**Methods:**

After stomach mobilization, gastric conduit formation, esophagus mobilization and two-field lymphadenectomy, the first robotic hand-sewn purse-string suture was applied to the esophageal muscular layer with an adequate margin above the tumor. A longitudinal incision in the anterior wall of the esophagus was made and the circular stapler anvil was inserted. The esophagus was transected by scissors 1 cm caudal to the first purse-string suture and the purse-string tied to secure the anvil. Then the second robotic hand-sewn purse-string suture was applied to the whole-layer of the proximal end of the esophagus and tied. Finally, the anvil was connected to the body of the stapler and fired.

**Results:**

The clinical data of ten patients who underwent robotic Ivor Lewis esophagectomy with an intrathoracic circular-stapled end-to-side anastomosis from February 2022 to April 2022 were collected. There were seven male and three female patients and had a mean age of 63.2 ± 7.6 years. Tissue donuts were complete in all cases and all operations were successfully performed without conversions. The mean overall operative time was 358.2 ± 40.3 min. The mean estimated blood loss was 83.2 ± 15.6 ml. The median length of hospital stay was 11.5 ± 4.1 days. All the patients had an uneventful postoperative period.

**Conclusion:**

Two purse-string sutures are necessary to obtain a tight seal of the esophageal tissue around the anvil to avoid potential anastomotic leak and are an essential process for the safety of circular-stapled anastomosis during robotic Ivor Lewis esophagectomy.

## Introduction

With the increased number of adenocarcinoma of the lower esophagus and cardia in the last decades (1), Ivor Lewis esophagectomy is increasingly being used (2). However, intrathoracic anastomosis is a technically difficult operation during totally minimally invasive Ivor Lewis esophagectomy. Creating a reliable esophagogastric anastomosis is essential to reduce the risk of leakage and related complications. Although various modified anastomotic techniques are developed to improve the quality of intrathoracic anastomosis after esophagectomy, anastomotic leakage remains the predominant surgical complication following esophagectomy, and the optimal technique for intrathoracic anastomosis after esophagectomy is still unclear (3–7).

The safety and feasibility of robot-assisted esophagectomy have been indicated (8, 9). With the advantage of the magnified vivid three-dimensional image, articulating forceps and tremor filtering, surgical robots facilitate the process of purse-string suture and ease insertion of the anvil into the esophagus stump (10). This study aimed to present our technical aspects and initial results of robotic Ivor Lewis esophagectomy using two purse-string sutures for circular-stapled anastomosis.

## Materials and methods

### Patients

From February 2022 to April 2022, the clinical data of ten consecutive patients with esophageal cancer of the lower esophagus or gastroesophageal junction were collected. All patients underwent robot-assisted (da Vinci Si robotic system) Ivor Lewis esophagectomy and two-field lymph node dissection in the Department of Thoracic Surgery, West China Hospital, Sichuan University, China. The reconstruction was performed with a gastric conduit and end-to-side circular-stapled anastomosis. All the patients were preoperatively diagnosed by upper digestive endoscopy and pathology. The study was approved by the ethics committee of our hospital and written informed consents were obtained from all of the patients.

### Operative procedure

General anesthesia and double-lumen endotracheal intubation were used. During the abdominal stage, the patients were placed in the supine position. Five trocars were inserted. Ports placement were described in our earlier report (10, 11). In detail, a 12 mm trocar for the camera was inserted just below the umbilicus, a 12 mm trocar for the assistant was placed at the right anterior axillary line below the costal arch, an 8 mm trocar for robotic arm 3 was placed at the left anterior axillary line about 2 cm below the costal arch, and an 8 mm trocar for robotic arm 2 and an 8 mm trocar for robotic arm 1 were placed at the left and the right mid-clavicular line about 2 cm above the umbilicus plane, respectively. The ports were docked to the patient cart which comes from the patient's head. Harmonic scalpel, fenestrated bipolar forceps, cardiere grasper, and needle drivers were used during the abdominal part. CO_2_ insufflation with 13 mmHg of pressure was used. After completion of stomach mobilization and abdominal lymph nodes dissection, a 4 cm-wide gastric conduit was intracorporeally fashioned using gold cartridges of 60 mm linear stapler (ECHELON FLEX™ Powered ENDOPATH^®^ Stapler, Johnson and Johnson Company, New Brunswick, NJ, United States). The upper part of the gastric fundus was left undivided, which facilitated delivering of the conduit into the right thorax. The staple line was over-sewn with barbed suture (Stratafix Sporal 3/0, Ethicon Endo-surgery, United States). Pyloroplasty was not routinely performed.

During the thoracic phase, the patients were placed in the left semi-prone position. Five ports were created. Ports placement were described in our earlier report (11). In detail, a 12 mm trocar for the assistant was placed at the 7th intercostal space in the posterior axillary line, and a 12 mm trocar for the camera was inserted at the 6th intercostal space just below the scapula angle. Two 8 mm trocars for robotic instruments were inserted: one for robotic arm 1 in the 4th intercostal space anterior to the scapula, and one for robotic arm 2 in the 8th intercostal space posterior to the posterior axillary line. The patient cart was docked onto the ports from the dorsocranial side. Fenestrated bipolar forceps, permanent monopolar cautery hook, monopolar scissors, and needle drivers were used during the thoracic part. CO_2_ insufflation with 8 mmHg of pressure was used. The azygos vein was routinely ligated and divided. The thoracic esophagus was mobilized to the apex of the chest and mediastinal lymph nodes were dissected. Then the patient cart is temporarily undocked from the patient.

**Figure 1:**
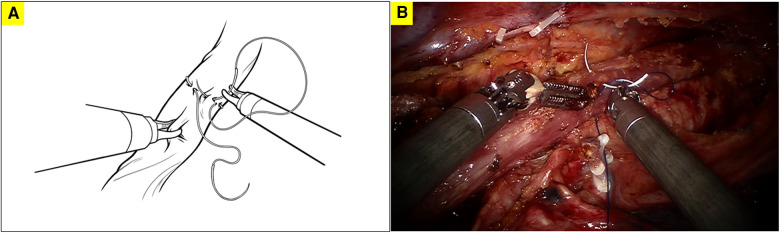
The first robotically sewn purse-string suture of the muscular layer of the esophagus; **A**. diagram illustrating the first robotically sewn purse-string suture; **B**. surgical picture of the first robotically sewn purse-string suture.

**Figure 2:**
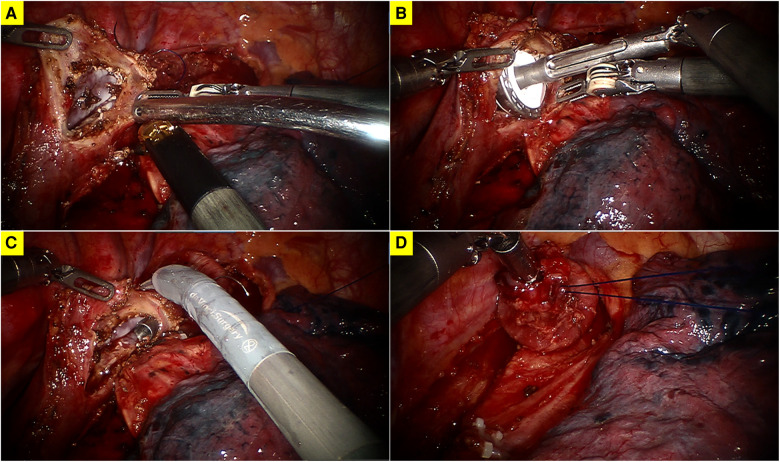
(**A**) A longitudinal incision at the anterior wall of the esophagus; (**B**) insertion of the anvil of a 25 mm stapler; (**C**) transection of the esophagus; (**D**) the fixation of the anvil.

**Figure 3:**
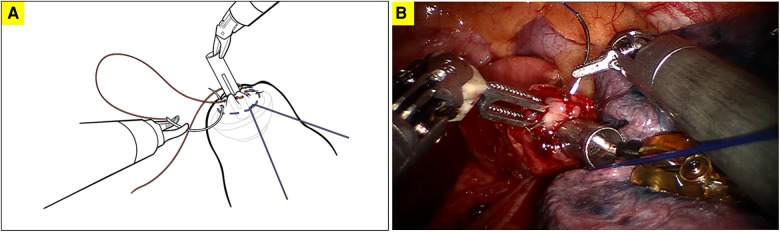
The superficial second purse-string suture of the full-layer of the proximal esophageal stump; **A**. diagram illustrating the second robotically sewn purse-string suture; **B**. surgical picture of the second robotically sewn purse-string suture.

The anastomosis was usually constructed above the level of the azygos vein. The patient cart was docked again from the patient's head. Trocar for the camera was placed at the 8th intercostal space posterior to the posterior axillary line. Trocar for robotic arm 1 was inserted at the 7th intercostal space in the posterior axillary line. Trocar for robotic arm 1 was inserted at 10th intercostal space in the subscapular line. The first robotically sewn purse-string suture of the muscular layer was performed using a 2–0 prolene suture (Figures 1A, B). The port at the 4th intercostal space was extended to about 4 cm and a wound protector was placed to introduce the circular stapler and remove of the specimen. Afterward, a longitudinal incision was made at the anterior wall of the esophagus about 2 cm below the planned anastomotic level (Figure 2A). The anvil of a 25 mm stapler was introduced into the lumen of the proximal esophagus (Figure 2B). The esophagus was transected by scissors 1 cm caudal to the first purse-string suture (Figure 2C). The distal esophagus was tied to prevent spillage of enteric contents. The anvil was secured with the first purse-string suture (Figure 2D). In order to prevent the esophageal edges from falling out of the purse-string suture, a superficial second purse-string suture of the full-layer of the proximal esophageal stump was performed using another 2–0 prolene suture (Figures 3A, B). The second purse-string was tied tight and the proximal esophageal edge was folded around the anvil's shaft.

The gastric conduit was pulled up, the specimen was removed, and the circular stapler was then inserted into the gastrostomy at the tip of the gastric conduit with the spike advancing out along the greater curvature just proximally to the gastroepiploic arcade. The anvil was mated with the stapler and fired. The proximal end of the gastric conduit was transected with a linear stapler and it was over-sewn with barbed suture. No patient needed to receive interrupted sutures to reinforce the anastomosis. Hemostasis was checked. A nasogastric tube and chest drainage tubes were inserted.

## Results

Between February 2022 and April 2022, a total of ten patients with esophageal cancer underwent the described procedure. There were seven male and three female patients and had a mean age of 63.2 ± 7.6 years. Tissue donuts were complete in all cases and all operations were successfully performed without conversions. The mean overall operative time was 358.2 ± 40.3 min. The mean estimated blood loss was 83.2 ± 15.6 ml. The median length of hospital stay was 11.5 ± 4.1 days. All the patients had an uneventful postoperative period and no anastomosis-related complications were observed. Six patients were diagnosed with squamous cell carcinoma of the lower esophagus and four patients were diagnosed with adenocarcinoma of the gastroesophageal junction. The postoperative pathologic examination revealed no tumor residual in the resection margin of the esophagus or stomach.

## Discussion

Anastomotic leakage contributes to a large number of perioperative morbidity and mortality following esophagectomy (12). The anastomotic technique is one of the variables that may influence the anastomotic safety. It is reported that circular stapler is less time-consuming and requires of less surgical expertise compared with the hand-sewn technique (13, 14). It is widely used for esophagogastric anastomosis after esophagectomy.

Sometimes, incomplete “donuts” were found during our single purse-string suture procedure. Then, interrupted sutures must be applied to reinforce the anastomosis, which was technically difficult and time-consuming. With the aim to obtain a tight seal of the esophageal tissue around the anvil to avoid incomplete “donuts” and create a reliable anastomosis, the double purse-string technique has been performed by our surgical team since February 2022. With the help of surgical robot, the presented anastomotic technique was easily reproducible and required minimal instrumentation of the esophageal remnant in comparison with the earlier double purse-string technique (7). Based on the preliminary results, this modified robotic technique was safe and feasible. It could prevent the esophageal edges from falling out of the purse-string suture and construct a reliable intrathoracic anastomosis after esophagectomy.

Although two purse-string sutures were conducted for our modified anastomotic technique, the total surgical time of our cohort was acceptable. With the help of the surgical robot, purse-string suture is easy to perform and the time taken for the second purse-string suture was only 7–10 min. The total surgical time was comparable to that of conventional robot-assisted circular-stapled anastomosis (7, 15). During the process of purse-string suture and fixation of the anvil, no surgical maneuver was performed at the planned level of anastomosis and didn’t lead to tissue tearing. Based on our experience, performing a second purse-string suture of the full-layer of the proximal esophageal stump can obtain a tight seal of the esophageal tissue around the anvil to avoid incomplete “donuts”. Performing a second purse-string suture was much easier than interrupted sutures applied to reinforce the anastomosis when incomplete “donuts” were found.

Various modified anastomotic techniques have been developed to improve the quality of the intrathoracic anastomosis after esophagectomy. For the transoral ORVIL technique, the esophagus was transected using a linear stapler, the point where the transverse and circular staple lines cross representing a weak spot within the anastomosis. Besides, the transoral ORVIL techniques required special assistance from the anesthetist. For reverse-puncture anastomosis, the increased length of the anvil resulting from the use of the reverse-puncture head required a relatively long proximal esophageal stump, raising the possibility of insufficient esophageal dissection. Additionally, the anvil's sharp tip increased the risk of esophageal stump injury during the operation (3).

There are many other risk factors which may lead to the failure of esophagogastric anastomosis except for anastomotic technique (12). First, a good vascular perfusion of the gastric conduit is important for anastomotic healing. A gastric conduit with width of 4–5 cm is associated with sufficient intramural vascular network (16, 17), which may result in good blood circulation at the tip of the gastric conduit. In order to guarantee perfect vascularization, we preferred to create a 4 cm wide gastric conduit during our procedure. Second, tension of the anastomosis is another risk factor of anastomotic leakage after esophagectomy (5). Intrathoracic anastomosis of Ivor Lewis esophagectomy needs a shorter gastric conduit in comparison with that of McKeown esophagectomy. A tension-free esophagogastric anastomosis is a quality indicator during our surgery. Third, the anastomotic site of the gastric conduit is a factor that may influence anastomotic leakage. Lai et al. reported that the defects of the stomach wall caused by anastomosis might have negative effects on blood supply to the area distal to anastomosis (18). In addition to create a wide gastric conduit, the incision in the gastric conduit for creating the anastomosis was made as far as possible from the longitudinal staple line and near the omentum (18, 19). There are many other risk factors for anastomotic leakage after esophagectomy, such as gastric conduit trauma and radiation to the anastomotic region (20, 21). Besides, higher body mass index, history of cardiac arrhythmia, an ASA grade of III or IV, tumor of the proximal esophagus, cervical anastomosis, diabetes mellitus, and chronic obstructive pulmonary disease are also predictors for anastomotic leakage after esophagectomy (12, 22, 23).

In summary, we present a modified robotic intrathoracic anastomotic technique after esophagectomy, which may improve the quality of the intrathoracic robotic anastomosis. Despite the small series, we believe our modified technique looks to be safe and reproducible. The double purse-string suture technique is useful to decrease the anastomotic leak rate after robotic Ivor Lewis esophagectomy. Further prospective controlled study with follow-up data is needed to evaluate the surgical outcomes achieved with this approach.

## Data Availability

The raw data supporting the conclusions of this article will be made available by the authors, without undue reservation.
